# Planned Retirement Timing in Europe: Are Europeans Adapting to the Policy of Extending Working Lives

**DOI:** 10.3389/fsoc.2021.691066

**Published:** 2021-08-05

**Authors:** Moritz Hess, Laura Naegele, Lena Becker, Jana Mäcken, Wouter De Tavernier

**Affiliations:** ^1^ Hochschule Niederrhein, University of Applied Sciences, Mönchengladbach, Germany; ^2^ Department of Ageing and Work, Institute for Gerontology, University of Vechta, Vechta, Germany; ^3^ SOCIUM Research Center on Inequality and Social Policy, University of Bremen, Bremen, Germany; ^4^ University of Cologne, University Hospital, Cologne, Germany; ^5^ KU Leuven, Center for Social and Psychology, Leuven, Belgium

**Keywords:** population ageing, preferred retirement age, retirement transition, retirement behaviour, SHARE, pension

## Abstract

As populations are ageing concerns regarding the sustainability of European welfare states have come to the forefront. In reaction, policy makers have implemented measurements aimed at the prolongation of working lives. This study investigates weather older workers have adapted their planned retirement age, as a result of this new policy credo. Based on data from Survey of Health, Ageing and Retirement in Europe (SHARE) the analysis shows an increase of the planned retirement age (1.36 years) across all ten European countries investigated, albeit with country-specific variations. Variations on the individual level can be detected in regard to gender, education and self-reported health status.

## Introduction

Population ageing with its increasing number of older people has resulted in concerns that the financial future of many European welfare states is in jeopardy since the late 1980s (Harper, 2015). The fear is that welfare expenditures for a growing number of older people have to be financed by contributions from a shrinking number of younger people. These concerns are raised with regards to the sustainability of health care, long-term care as well as pension systems. Concerning the latter, particularly, public pay-as-you-go pensions are seen as vulnerable to population ageing (Ebbinghaus and Naumann, 2020).

In response policy makers in many European countries have implemented reforms aimed at delaying retirement and extending working lives. They have increased state pension ages, closed early retirement options and invested in the employability of older workers (De Tavernier et al., 2019). It seems that these reforms have taken effect as actual retirement age as well as older workers’ employment rates are increasing; however, from different starting points and with different paces (Ebbinghaus and Hofäcker, 2013). Yet, many of these reforms have a time-lagged effect as for example increases of state pension ages are realized in a stepwise process (Hofäcker, 2015). Thus, today's pensioners have often not felt the full impact of the reforms (yet).

Nevertheless, previous studies have shown that older workers not only adapt due to the new credo of extended working lives, but are also quite precise when estimating their retirement timing. Older workers’ planned retirement age might therefore help evaluate the reforms' impact and could be used as a proxy for older workers' future actual retirement behavior (Haider and Stephens, 2007; Örestig et al., 2013). Hess (2017a) found that the preferred retirement—the age at which one wants to retire—has increased in several European countries. Single country studies find that the expected or planned retirement age—the age at which one realistically believes to retire—has increased in Germany (Coppala and Wilke, 2010), the Netherlands (De Grip et al., 2013), Sweden (Örestig et al., 2013) as well as the United States of America and Australia (Sargent-Cox et al., 2012). So far however, no studies exist that investigates the planned retirement age and its potential changes in a European country-comparative perspective. The study at hand aims to fill this gap in the literature.

Based on data from Survey of Health, Ageing and Retirement in Europe (SHARE) we, thus, explore the planned retirement age in a cohort and country comparison. Further analyses consider country, socio-economic status, and gender differences. In the following we will first discuss the societal contexts in more detail and present and derive our research questions.

## Societal Contexts and Research Questions

Increasing life expectancy and decreasing fertility rates are resulting in a growing number of older people in absolute and relative terms. By the year of 2070 30% of all Europeans are estimated to be aged 65 and older, up from about 20% today (European Commission, 2020). Simultaneously, in the years to come, the workforce potential—meaning the share of the working-age population—will decline in most European countries (van der Gaag and de Beer 2014). As many of Europe’s public pension systems are based on the pay-as-you-go principle this means that fewer and fewer workers paying contributions to the pension system are facing more and more retired people receiving pensions. In addition, due to an increased life expectancy, these retired people do live longer (Hofäcker et al., 2015).

This development has increased concerns about the long-term financial sustainability of public pension systems. Harper (Harper 2015, 23) summarizes this concisely: “the social security systems now face serious financing problems as the number of beneficiaries is increasing at a time when the working population is declining—a simultaneous increase in payments and decrease in revenues”. In some European countries—in particular with conservative welfare states—the challenges stemming from population ageing seem even more problematic as the policy of early retirement has led to comparably low retirement ages. Based on the idea of the lump-of-labor and with the aim of decreasing unemployment rates the policy of early retirement offered older workers financially attractive early retirement options, allowing them to exit the labor market well before the state pension age with only small pension reductions (Hess et al., 2016). This resulted in a disadvantageous ratio of retired over working population.

In response policy makers in Europe have implemented reforms aimed at extending working lives by delaying retirement timing and, hence, decreasing the number of people receiving pensions and increasing the number of people paying contributions. Additionally, in some countries, selected sectors—high-technology, health and care as well as crafts sector—are faced with an increasing shortage of skilled workers (Brunello and Wruuck, 2019; Ehrlich et al., 2020; Naegele 2020). Sustaining experienced and reliable older workers is considered one measure to mitigate this lack of skilled workers and to ensure companies’ competitiveness in the future.

These reforms included the closing of early retirement options or making them financially much less attractive, and, consequently, making early retirement more expensive for older workers (Hofäcker 2015). State pension ages were raised and in some countries are even indexed to life-expectancy (Naegele and Bauknecht 2019). Furthermore, public pensions were lowered and privatization and marketization elements were introduced to compensate the lower incomes from the public pensions (Ebbinghaus 2016, 2021). Efforts were also made to strengthen older workers’ workability and employability. Fighting ageism at the workplace and creating age-inclusive company cultures became more important in the opinion of policy makers and employers (Naegele et al., 2018, 2019). Investments in measures of life-long learning and training for older workers have increased and the concept of a life-course oriented human-resource strategy emphasized the importance of preventive health care programs at the workplace (Frerichs et al., 2012; Walker, 2019). The effort at the national and company level are supported by international organizations like the Organization for Economic Co-operation and Development (OECD) (Boppel et al., 2011) and the European Union (EU) (Walker and Maltby 2012). In particular the role of the EU’s Open Method of Co-ordination and its pension sustainability target to increase the employment rate of the 55–64-years-old to 50% or more played a crucial role (Allaj et al., 2016). It seems as if the reforms have been effective as older workers’ employment rates and retirement ages are increasing: “Labour market participation at older ages has substantially increased since the turn of the century and companies are using this new potential—employment rates of older workers have risen much more strongly than for the rest of the population and this rise is marked across all education levels.” (OECD 2020, 39).

It must, however, be mentioned that the reforms aimed at delaying retirement are not the only potential explanation for these developments (Hess 2018). The overall labour market development has been well and the demand for workers has increased—at least in some European countries. In addition, today’s older workers are healthier and better educated than their predecessors (Hess 2016).

Against this background, the main aim of the paper is to explore the influence of the above-described developments and policy measurements on the planned retirement age of older workers. Consequently, the first research question is: How has the policy shift towards extending working lives affected older workers’ planned retirement age? Secondly, as the increase in the employment rates does differ between countries the paper also wants to investigate these country differences. Hence, the second research question is: Does the effect of the policy shift towards extending working lives on older workers' planned retirement age differ between countries? The third research question addresses potential group differences in this regard and states: Does the effect of the policy shift towards extending working lives on older workers' planned retirement age lives differ between social groups? In the following we give a brief overview of previous research, expand on the theoretical considerations we base our analysis on and develop our hypotheses.

## State of the Art and Theoretical Considerations

The study at hand is not the first to explore how older workers’ ideas when to retire have developed over time. Using data from the European Social Survey and Eurobarometer Hess (2017a) shows that the age at which older workers would like to retire has increased in the 12 European countries investigated. Although the preferred retirement age and the planned retirement age differ, it seems that older workers are reacting to the new credo of late retirement, albeit with group-specific differences. Whereas De Grip et al. (2013) show for the Netherlands and Coppala and Wilke (2014) for Germany that higher educated seem to be more responsive (and adjust more) to policies promoting the extension of working lives, Öresting et al. (2013) find differences in regard to retirement preferences between men and women and those in favorable and unfavorable working conditions in Sweden. The latter, as the authors argue, finding themselves often in occupational positions with high psychological and physical strains, resulting in earlier retirement preferences. Sargent-Cox et al. (2012) report similar effects for poor health and an overall younger expected retirement age for Australian workers compared to the U.S. sample in the study. With the exception of Hess (2017a) these studies have either taken on a single country focus (Örestig et al., 2012; De Grip et al., 2013; Coppola and Wilke, 2014) or investigated the preferred retirement age from a comparative perspective at only one point in time (Hofäcker 2015; Sargent-Cox et al., 2012). The study at hand will add to the existing research corpus by taking on a country comparative and time-sensitive perspective when investigating the development of the planned retirement in Europe.

The aim of this study is to link the trend towards extending working lives on the country level (macro) and the workplace level (meso) with the planned retirement age on the individual level (micro). Institutionalism offers such a link, as it not only explains “how opinions, preferences, actions and outcomes are constituted and related to norms and policies, but also how they are interrelated” (De Tavernier, 2016, 7). Institutionalism can be divided into three different strands (rational choice, sociological and historical institutionalism), all with different emphases. Whereas rational choice institutionalism focusses on the individual rational actor that executes his or her preferences, sociological institutionalism explains these preferences as a result of internalized societal norms as well as one’s individual identity. Historical institutionalism incorporates the temporal aspect by addressing (re-)production and the occasional change of policies and norms over time (De Tavernier, 2016). For the paper at hand we focus on sociological institutionalism as theoretical basis as it emphasizes the importance of cultural values and norms, reflected in the above-mentioned policy shift towards the prolongation of working lives, for an individual’s retirement planning (Hall and Taylor, 1996; Knill and Lenschow, 2001).

According to sociological institutionalism institutions shape individuals’ action with incentives and constraints for which they set a frame, which aligns to commonly shared norms and values. Sociological institutionalism, thus, is making the explicit link between individual actions and cultural contexts (Hall and Taylor, 2007; Bevir and Bevir, 2010). Jensen (1996), for example, shows that shared norms and values have an influence on whether individuals use certain offers of welfare state institutions at all, while Pfau-Effinger (2005) and Hall and Taylor (2007) point to the importance not only of internalized values and norms, but also of social role expectations expressed through institutional frameworks. The argument of sociological institutionalism is not deterministic in nature; rather, it is about limiting the options from which the individual can choose: “Institutions influence behavior not simply by specifying what one should do but also by specifying what one can imagine oneself doing in a given context” (Hall and Taylor, 2007). Elaborated upon earlier work, Hall and Taylor (1996) even went so far to hold institutions responsible, not only to simply affect "the strategic calculations of individuals, as rational choice institutionalists contend, but also their most basic preferences and very identity” (Hall and Taylor, 1996, 948).

Taking a sociological institutionalism perspective on the questions of the study at hand, one could make the following argument: the reforms aimed at extending working lives have changed the institutional level in a way that it now incentives later retirement and restricts behaviors (e.g. early retirement) that are inconsistent with the (newly) shared social norm of prolonged working lives. This should affect older workers and lead to an increased planned retirement age. Hence, our first hypothesis (H1) is: the reforms aimed at extending working lives have resulted in an increase of the planned retirement age of older workers in the countries investigated. As retirement preferences are not only a result of being embedded in institutional context, but also of an individual’s experiences and characteristics, exploring social group differences seems purposeful. Regarding retirement planning earlier research has pointed towards effects of gender, health, income level and educational differences (Hofäcker et al., 2015; Esser 2005; Stiemke and Heβ, 2020). Therefore, our second hypothesis (H2) states: the increase of planned retirement age differs between social groups. Previous research produced ambivalent results regarding the direction of these group differences (see for example Coppola and Wilke 2014; Hess 2017b), hence, we do not predict for which groups the increase is stronger and for which groups it might be weaker. As the often so called “paradigm shift” in labour market policies—from early retirement to extended working lives—might be mediated by specific labour market and pension as well as cultural settings and country-specific age norms (Hess 2017a), taking on a country-comparative perspective appears necessary. Thus, our third hypothesis (H3) is: the increase of the planned retirement age differs between countries. Again, we make no prediction about the direction and extend of these differences in the increase. In the following sections of the paper the methods used are introduced, results are presented and discussed.

## Methods

To explore our research questions, we use data derived from the SHARE. SHARE is a longitudinal panel survey in which people who are 50 years and older are interviewed about their health, household composition, economic situation, as well as work, volunteering and physical activity. Interviews are conducted biannually in a variety of European countries (Börsch-Supan et al., 2013). The sample for the analysis at hand is restricted to those in employment and aged between 55 and 65. We combine those from wave 1, which was collected in 2004, and wave 6—collected in 2015 to allow for a comparison over time. Hence, the sample was further restricted to countries that participated in wave 1 and wave 6. These countries include: Austria, Germany, Sweden, Spain, Italy, France, Denmark, Greece, Switzerland, and Belgium. The sample consists of 15,441 respondents. Country-specific case numbers range from *n* = 593 in Italy to *n* = 2,566 in Belgium.

The planned retirement age was operationalized using the question on the expected age of first collection of pensions. Answers were given in years. Outliers (less than 3 percent) under 55–65 years old were deleted. To test the first hypothesis that the planned retirement age has increased over time regression technique was employed. The planned retirement age served as dependent variable and hence, linear regressions were used. Independent variables were the cohort (2004 vs 2015), gender (female vs male) and level of education. Country-specific educational categories were classified according to the International Standard Classification of Education (ISCED-97) (UNESCO 2012) and recoded into low (ISCED 1, 2), medium (ISCED 3, 4) and high (ISCED 5, 6). In addition, interaction effects between cohort on the one hand and gender and education on the other hand were included in the models. Control variables were age, self-rated health, the ability to make ends meet and the country of residence. Self-rated Health and the ability to make ends meet were operationalized as dichotomous variables with (poor vs good self-rated health) and (problems to make ends meet vs no problems to make ends meet). The control variables were chosen based on findings from previous literature (see for example Hofäcker, 2015).

## Results

As shown in Table 1 the distribution of the sample is as expected: About half of the sample are women and the average age is between 56 and 58. About half of the respondents report poor health and about a quarter state they have problems to make ends meet. These variables do not vary substantially between 2004 and 2015. One can observe that the share of older workers with low education is decreasing and, thus, the share of those with medium and high education is increasing. This is in line with timing of the educational expansion (Breen 2010) from which the 2015 cohort of older workers did probably already benefit. The variable of main interest - planned retirement age - has increased by 1.36 years from 63.11 to 64.47. This development is in line with the first hypothesis.

**TABLE 1:** Descriptive overview of sample.

	Cohort 2004			Cohort 2015			∆ 2004 and 2015
	Obs	Mean/Share	Std. Dev	Obs	Mean/Share	Std. Dev	Mean
Planned retirement age	6,767	63.11	2.88	8,674	64.47	2.45	1.36
Female	6,767	49%	0.5	8,674	53%	0.5	0.04
Age	6,767	56.22	4.28	8,674	57.58	4	1.36
Poor self-rated health	6,767	55%	0.5	8,674	56%	0.5	0.01
Problems to make ends meet	6,767	28%	0.45	8,674	25%	0.43	-0.03
Low education	2,181	32%	0.47	1,620	19%	0.39	-0.13
Medium education	2,664	39%	0.49	3,853	44%	0.5	0.05
High education	1922	28%	0.45	3,201	37%	0.48	0.09

Table 2 shows the variation across the countries and the two cohorts. In general, higher planned retirement ages are found in the Scandinavian countries—which is in line with Hess (2017a)—and the lowest in Austria. In addition, it demonstrates that the increase of the planned retirement ages is a pan-european development that is taking place in all ten countries included in the analysis and is not driven by certain outliers. The strongest increase is found in Italy and the weakest in Spain. This country variation was expected in our third hypothesis.

**TABLE 2:** Overview of planned retirement age country values.

	Cohort 2004			Cohort 2015			∆ 2004 and 2015
	Obs	Mean	Std. Dev	Obs	Mean	Std. Dev	Mean
Austria	231	61.16	2.9	437	61.85	2.7	0.69
Germany	1,042	63.44	2.2	1,462	64.67	1.7	1.23
Sweden	1,220	64.40	2	712	64.91	1.6	0.51
Spain	346	63.93	2.1	514	64.38	1.9	0.45
Italy	361	61.01	3.5	232	64.86	3	3.85
France	822	60.61	2.7	739	62.12	2.5	1.51
Denmark	878	65.14	0.9	1,571	66.48	1.4	1.34
Greece	386	63.25	3.8	650	64.03	3.5	0.78
Switzerland	422	63.63	1	850	64.46	1	0.83
Belgium	1,059	62.16	3.1	1,507	64.05	2.4	1.89

Table 3 depicts the results from the linear regressions. The findings regarding the control variables are in line with previous research. Those with poor self-rated health plan to retire earlier than those with self-reported good health (Nilsson et al., 2016; Hess and Naegele, 2020). This might be explained by the possibility to use different early retirement options that are connected to one’s health status (for example disability pensions). Those with difficulties to make ends meet in contrast plan to retire later. Hess (2017b) finds the same correlation with German data and explains it by financial pressure that can be mitigated with delayed retirement and consequently higher pensions benefits. The positive association between age and planned retirement timing is line with results of Hofäcker et al. (2015).

**TABLE 3:** Linear regressions with planned retirement age as dependent variable.

	Coefficient	*p*-value
*Cohort (Ref: 2004)*		
2015	0.993^a^	(0.000)
*Gender (Ref: Male)*		
Female	−0.328^a^	(0.000)
*Education (ref: low education)*		
Medium	−0.209^b^	(0.002)
High	0.133	(0.069)
*Age*		
In years	0.058^a^	(0.000)
*Self-rated health (Ref: Good)*		
Poor	−0.209^a^	(0.000)
Problems to make ends meet (ref: Not)		
Yes	0.302^a^	(0.000)
*Country (Ref: Germany)*		
Austria	−2.649^a^	(0.000)
Sweden	0.573^a^	(0.000)
Spain	−0.082	(0.380)
Italy	−1.486^a^	(0.000)
France	−2.670^a^	(0.000)
Denmark	1.671^a^	(0.000)
Greece	−0.734^a^	(0.000)
Switzerland	−0.150	(0.060)
Belgium	−0.923^a^	(0.000)
*Interaction Cohort and Education*		
2015 # medium	0.163	(0.085)
2015 # high	0.124	(0.215)
*Interaction Cohort and Gender*		
2015 # gender	0.206^c^	(0.005)
*Constant*	60.470^a^	(0.000)
Observations *R* ^2^	15,4410.31	

Levels of Significance.

c *p* < 0.05.

b *p* < 0.01.

a *p* < 0.001.

When looking at our hypotheses, the increase in the planned retirement age between 2004 and 2015 is supporting hypothesis 1. This result is also reflected for European data in Hess (2017a) as well as in the earlier discussed studies with specific country focusses on Germany (Coppola and Wilke, 2014), Netherlands (De Grip et al., 2013), Sweden (Öresting et al., 2013) and Australia/U.S. (Sargent-Cox et al., 2012). It seems as if the reforms aimed at delaying retirement have also affected prospective retirees and their plans when to make the transitions from work to retirement.

Supporting hypothesis 2 we find that planned retirement age does significantly vary by gender and education. In line with previous research (De Preter et al., 2013; Holman et al., 2020) women plan to retire earlier than men, probably due to the lower labor market attachment of women, certain early retirement options only accessible for women and couples retiring together whereas women on average are somewhat younger than their partners. For education the regression shows a u-shaped connection. Those with medium education plan to retire earlier than those with low and high education. This u-shaped connection was found earlier with German data for the actual retirement age by Hofäcker et al. (2015) and for the expected retirement age by Hess (2018). “However, while high-skilled workers both want and expect to retire late, low-skilled workers prefer to retire early but expect that they have to work longer in order to ensure a reasonable pension. This finding hints at rising social inequality in the transition from work to retirement” (Hess 2018, 1). We also find that the increase of the planned retirement age is stronger for women than for men indicated by the positive interaction effect of cohort and gender. This is in line with steeply rising female employment rates among older workers (Ebbinghaus and Hofäcker, 2013). No significant interaction effect for cohort on the one hand and education on the other hand was found.

## Discussion

The demographic ageing has fueled concerns and started discussions about the long-term financial sustainability of welfare states as a whole and for pay-as-you-go public pension systems in particular. Policy makers have reacted by implementing reforms aimed at delaying retirement and extending working lives. These reforms include among others the increase of statutory state pension ages, the closing of early retirement options and investments in older workers’ employability and workability. It seems as if the reforms are being effective as older workers’ employment rates are increasing. The study at hand explored if these increases are mirrored in the planned retirement age of older workers. Based on date from SHARE and comparing the years 2004–2015 we find an average increase of 1.36 years. This increase can be observed in all analyzed countries, however with large variation in the extent. In addition, the increase is stronger for women then for men narrowing the gap between women and men.

When interpreting the results at least four limitations must be acknowledged. Alternative explanations besides the reforms aimed at extending working lives might be found for the increase of the planned retirement age. As earlier research has shown retirement decisions—and the planning that goes along with them—are multi-factored and complex. It could be argued that the general good development of the labor market in some countries has influenced the detected increase. In addition, cohort effects might play a role. Today’s older workers are better educated and healthier than their predecessors, in particular more women are working in these cohorts. Hence, the fact that increasingly more women enter and “age” in the labour market might be reflected in the observed stronger increase of the planned retirement age for women. The second limitation is that a potential effect of the economic and financial crisis from 2008 on the planned retirement age is not controlled for in the paper. The strong increase of the planned retirement age in Italy supports such an effect, as older workers in Italy might have come under pressure to ensure sufficient pensions due to job-loss and the overall economic downfall in the crisis. However, the weak effect in Greece contradicts this assumption, highlighting the necessity of further, more in-depth investigations of the main drivers behind the increase of planned retirement age. The third limitation is the limited number of countries, which should be addressed in future research. In particular, Central and Eastern European countries would be an interesting addition to the analysis. Finally, a potential selection bias must be acknowledged: Only those still in employment at the age of 55–65 can report a planned retirement age; those who are retired or otherwise inactive cannot and they might differ from those who are employed systematically. However, previous research investigating expected and preferred retirement age in a cohort comparison (Hess 2017a, 2018) found that this bias does not contort the results, when conducting a Heckman Test.

Despite these limitations, the study makes an important contribution and extends previous research in the field. It is the first analysis that shows that the planned retirement is increasing in many European countries. This might be seen as some sort of relief for policy makers, but these findings also hint at the rise of social inequalities in retirement transitions. Should it appear that certain social groups are disadvantaged with regard to their retirement planning, policy makers and scientist alike need to take notice and take action to prevent inequalities from exacerbating.

## Data Availability

Publicly available datasets were analyzed in this study. This data can be found here: http://www.share-project.org/home0.html.
